# Efficacy and safety of fondaparinux in preventing venous thromboembolism in Chinese cancer patients: a single-arm, multicenter, retrospective study

**DOI:** 10.3389/fonc.2023.1165437

**Published:** 2023-05-29

**Authors:** Lei Wang, Zhong Su, Chunying Xie, Ruijun Li, Wei Pan, Lu Xu, Fei Chen, Gang Cheng

**Affiliations:** ^1^ Department of Medical Oncology, The Afflicted Bozhou Hospital of Anhui Medical University, Bozhou, China; ^2^ Department of Oncology, Shandong Zouping People’s Hospital, Zouping, China; ^3^ Department of Oncology, The Second Affiliated Hospital of Nanchang University, Nanchang, China; ^4^ Department of Medical Oncology, Zhengzhou People’s Hospital, Zhengzhou, China; ^5^ Department of Radiation Oncology, The Affiliated Jiangning Hospital of Nanjing Medical University, Nanjing, China; ^6^ Department of Oncology, Yongkang First People’s Hospital, Yongkang, China; ^7^ Department of Oncology, The Central Hospital of Xiaogan, Xiaogan, China

**Keywords:** fondaparinux, cancer, venous thromboembolism, bleeding event, adverse event

## Abstract

**Objective:**

Fondaparinux is a synthetic anticoagulant for the prevention of venous thromboembolism (VTE), and its administration in Chinese cancer patients is rarely reported. This study aimed to assess the efficacy and safety of fondaparinux in preventing VTE in Chinese cancer patients.

**Methods:**

A total of 224 cancer patients who received fondaparinux treatment were reviewed in this single-arm, multicenter, retrospective study. Meanwhile, VTE, bleeding, death, and adverse events of those patients in the hospital and at 1 month after treatment (M1) were retrieved, respectively.

**Results:**

The in-hospital VTE rate was 0.45% and there was no (0.00%) VTE occurrence at M1. The in-hospital bleeding rate was 2.68%, among which the major bleeding rate was 2.23% and the minor bleeding rate was 0.45%. Moreover, the bleeding rate at M1 was 0.90%, among which both the major and minor bleeding rates were 0.45%. The in-hospital death rate was 0.45% and the death rate at M1 was 0.90%. Furthermore, the total rate of adverse events was 14.73%, including nausea and vomiting (3.13%), gastrointestinal reactions (2.23%), and reduced white blood cells (1.34%).

**Conclusion:**

Fondaparinux could effectively prevent VTE with low bleeding risk and acceptable tolerance in cancer patients.

## Introduction

Venous thromboembolism (VTE) is a disease of venous obstructive reflux disorder caused by venous thrombosis, which is relatively common in cancer patients with an incidence rate of approximately 20%, especially in cancer patients who received surgery and chemotherapy (both are risk factors of VTE), and notably aggravates the mortality of these patients (1–5). Generally, anticoagulants, such as low-molecular-weight heparin and apixaban, are used for the treatment of VTE in cancer patients (6–8), while the use of anticoagulants may lead to bleeding events in patients, which causes significant long-term disability and even death (9, 10). Therefore, it is crucial for cancer patients to find an effective and safe anticoagulant that can both prevent VTE and minimize the risk of bleeding events.

Fondaparinux is a synthetic heparin pentasaccharide, which inhibits thrombin production and the growth of thrombus by selectively inhibiting factor Xa (11, 12). As a type of anticoagulant for the prevention and treatment of VTE (13), fondaparinux has a series of advantages, such as high bioavailability, fast onset of action, and direct renal excretion, which is considered to be more effective and safer than heparin anticoagulants and vitamin K antagonists, such as unfractionated heparin, low-molecular-weight heparin, and warfarin (11, 14, 15). For example, a study shows that the incidence of VTE and major bleeding in cancer patients who received fondaparinux is lower than in cancer patients who received warfarin (14). In addition, another study also indicates that the rate of VTE is similar while the risk of major bleeding is reduced in cancer patients who received fondaparinux compared with cancer patients who received enoxaparin and unfractionated heparin (15). However, the rates of VTE, bleeding, death, and adverse events in Chinese cancer patients who received fondaparinux are rarely reported.

Therefore, this single-arm, multicenter, retrospective study aimed to evaluate the efficacy and safety of fondaparinux in preventing VTE in Chinese cancer patients.

## Methods

### Patients

A total of 224 cancer patients who received fondaparinux for the prevention of VTE from February 2021 to February 2022 were reviewed in this single-arm, multicenter, retrospective study. The screening criteria were as follows: (1) age over 18 years; (2) hospitalized patients; (3) confirmed as solid malignancies, which included but were not limited to lung cancer, esophageal cancer, stomach cancer, pancreatic cancer, colorectal cancer, liver cancer, breast cancer, gynecologic cancer, kidney cancer, bladder cancer, prostate cancer, testicular cancer, or lymphoma, with locally advanced or metastatic disease; (4) Eastern Cooperative Oncology Group Performance Status 0–2, or Karnofsky performance status 70–100; (5) received fondaparinux only for the prevention of VTE; and (6) had complete clinical data. Patients who met the following conditions were excluded: (1) diagnosis of deep venous thrombosis at admission (except for intramuscular venous thrombosis); (2) had a history of anticoagulant drugs within 72 h before admission; (3) platelet count <50 × 10^9^/L at admission; (4) had primary brain cancer, or a known history of metastatic brain cancer; (5) had other hematologic malignancies except for lymphoma; (6) underwent surgery (such as major orthopedic surgery, trauma surgery, spine surgery, eye surgery, and brain surgery) other than oncologic surgery 3 months before admission; (7) had a history of active bleeding, history of gastrointestinal ulcers, history of vasoproliferative gastrointestinal disease, history of hemorrhagic stroke, history of congenital or acquired bleeding disorders, or other high risk factors for bleeding; (8) had severe kidney impairment (creatinine clearance rate ≤20 ml/min); (9) pregnant or lactating female patients; and (10) any other conditions for which the investigator deemed the patients unsuitable for this study. The study was approved by the Ethics Committee.

### Medication

Fondaparinux was given subcutaneously at 2.5 mg/day for 7–10 days in the hospital or until discharge. For patients with a creatinine clearance rate of 20–50 ml/min, fondaparinux was given subcutaneously at 1.5 mg/day. For patients with medium or high risk for VTE (Khorana score ≥2), the administration of fondaparinux was recommended to be extended to 4 weeks. During the fondaparinux administration, antiplatelet and oncology drugs were used for appropriate patients.

### Data collection

Demographics, disease history, treatment history, disease characteristics, biochemistry indexes, and current treatment information were obtained. Moreover, VTE, bleeding (major bleeding and minor bleeding), and death in the hospital as well as at 1 month after treatment (M1) were obtained for evaluations. VTE was defined as the combination of symptomatic pulmonary embolism (PE), symptomatic deep vein thrombosis (DVT), asymptomatic DVT, distal DVT, and proximal DVT. Major bleeding was defined as the combination of fatal bleeding, significant bleeding (hemoglobin declined ≥2 g/dl, or leading to the transfusion of red blood cells in whole blood more than two units), and bleeding at a critical position (retroperitoneal, intracranial, spinal, intraocular, joint space, pericardial, compartment syndrome, etc.). Minor bleeding was defined as the combination of gum bleeding, nose bleeding, blood in the stool, other minor bleeding, etc. In addition, the adverse events of patients were also collected.

### Statistics

GraphPad Prism v9.0 (GraphPad Software Inc., USA) was adopted for figure plotting. Continuous data were described as mean and SD if normally distributed, and median and interquartile range (IQR) if not normally distributed. Count data were expressed as counts and percentages. Correlation of platelet count with bleeding occurrence was determined by the chi-square test. *p* < 0.05 was considered significant.

## Results

### Demographics, disease history, and treatment history in fondaparinux-treated cancer patients

In total, 224 cancer patients with a mean age of 60.77 ± 14.70 years were included in the study. There were 108 (48.21%) female and 116 (51.79%) male patients. The number of patients with a history of hypertension, diabetes, cerebrovascular disease, ischemic heart disease, central venous cannulation, vein thrombosis or PE, varicosity, acute myocardial infarct, and stroke was 40 (17.86%), 17 (7.59%), 9 (4.02%), 5 (2.23%), 5 (2.23%), 2 (0.90%), 1 (0.45%), 1 (0.45%), and 1 (0.45%), respectively. In addition, there were 132 (58.93%) patients with a history of tumor treatment and 20 (8.93%) patients with a history of anticoagulant treatment. More detailed information about the demographics, disease history, and treatment history of cancer patients is shown in **Table 1**.

**Table 1:** Demographics, disease history, and treatment history.

Items	Patients (N = 224)
Demographics	
Age (years), mean ± SD	60.77 ± 14.70
Sex, n (%)	
Female	108 (48.21)
Male	116 (51.79)
History of drinking, n (%)	19 (8.48)
History of smoking, n (%)	30 (13.39)
Disease history	
History of hypertension, n (%)	40 (17.86)
History of diabetes, n (%)	17 (7.59)
History of cerebrovascular disease, n (%)	9 (4.02)
History of ischemic heart disease, n (%)	5 (2.23)
History of central venous cannulation, n (%)	5 (2.23)
History of vein thrombosis or pulmonary embolism, n (%)	2 (0.90)
History of varicosity, n (%)	1 (0.45)
History of acute myocardial infarct, n (%)	1 (0.45)
History of stroke, n (%)	1 (0.45)
Treatment history	
History of tumor treatment, n (%)	132 (58.93)
Chemotherapy	116 (51.79)
Surgery	64 (28.57)
Radiotherapy	48 (21.43)
Targeted therapy	30 (13.39)
Immunotherapy	16 (7.14)
Intervention	5 (2.23)
History of anticoagulants, n (%)	20 (8.93)

SD, standard deviation.

### Disease characteristics, biochemical indexes, and current treatment information in fondaparinux-treated cancer patients

There were 66 (29.46%) gynecologic cancer patients, 46 (20.54%) lung cancer patients, 21 (9.38%) colorectal cancer patients, 17 (7.59%) nasopharyngeal cancer patients, 15 (6.70%) gastric cancer patients, 14 (6.25%) esophageal cancer patients, 9 (4.02%) liver cancer patients, 6 (2.68%) pancreatic cancer patients, 4 (1.79%) lymphoma patients, and 26 (11.61%) patients with other cancers in this study. The number of patients receiving chemotherapy, radiotherapy, targeted therapy, immunotherapy, palliative therapy, hormone therapy, and pain therapy was 122 (54.46%), 67 (29.91%), 43 (19.20%), 24 (10.71%), 8 (3.57%), 7 (3.13%), and 7 (3.13%), respectively. Furthermore, the median (IQR) duration of patients receiving fondaparinux was 9.00 (7.00–10.00) days. A total of 87 (38.84%) patients received successive fondaparinux administration. Additionally, there were 4 (1.79%) patients with a combination of aspirin. More specific information on the disease characteristics, biochemical indexes, and current treatment of the cancer patients is shown in **Table 2**.

**Table 2:** Disease characteristics, biochemical indexes, and current treatment information.

Items	Patients (*N* = 224)
Disease characteristics	
Cancer type, n (%)	
Gynecologic cancer	66 (29.46)
Lung cancer	46 (20.54)
Colorectal cancer	21 (9.38)
Nasopharyngeal cancer	17 (7.59)
Gastric cancer	15 (6.70)
Esophageal cancer	14 (6.25)
Liver cancer	9 (4.02)
Pancreatic cancer	6 (2.68)
Lymphoma	4 (1.79)
Others	26 (11.61)
TNM stage, n (%)	
I	10 (4.46)
II	29 (12.95)
III	89 (39.73)
IV	96 (42.86)
Khorana score, n (%)	
0	66 (29.46)
1	105 (46.88)
2	32 (14.29)
3	14 (6.25)
4	4 (1.79)
5	1 (0.45)
Not assessed	2 (0.90)
Biochemical indexes	
Hemoglobin, n (%)	
Normal	99 (44.20)
Abnormal	121 (54.02)
Not assessed	4 (1.79)
Platelet count, n (%)	
Normal	161 (71.89)
Abnormal	58 (25.89)
Not assessed	5 (2.23)
Serum creatinine, n (%)	
Normal	164 (73.21)
Abnormal	52 (23.21)
Not assessed	8 (3.57)
D-dimer, n (%)	
Normal	90 (40.18)
Abnormal	123 (54.91)
Not assessed	11 (4.91)
Current treatment	
Cancer therapy, n (%)	
Chemotherapy	122 (54.46)
Radiotherapy	67 (29.91)
Targeted therapy	43 (19.20)
Immunotherapy	24 (10.71)
Palliative therapy	8 (3.57)
Hormone therapy	7 (3.13)
Pain therapy	7 (3.13)
Fondaparinux duration (days), median (IQR)	9.00 (7.00–10.00)
Successive fondaparinux administration, n (%)	87 (38.84)
Combination of antiplatelet drugs, n (%)	
Aspirin	4 (1.79)

Normal values of hemoglobin were defined as 120–160 g/L for men and 110–150 g/L for women; normal values of platelet count were defined as 100–300×10^9^/L; normal values of serum creatinine were defined as 53–106 μmol/L for men and 44–97 μmol/L for women; normal values of D-dimer were defined as ≤0.55 mg/L.

TNM, tumor-node-metastasis; IQR, interquartile range.

### VTE rate in fondaparinux-treated cancer patients

**Figure 1:**
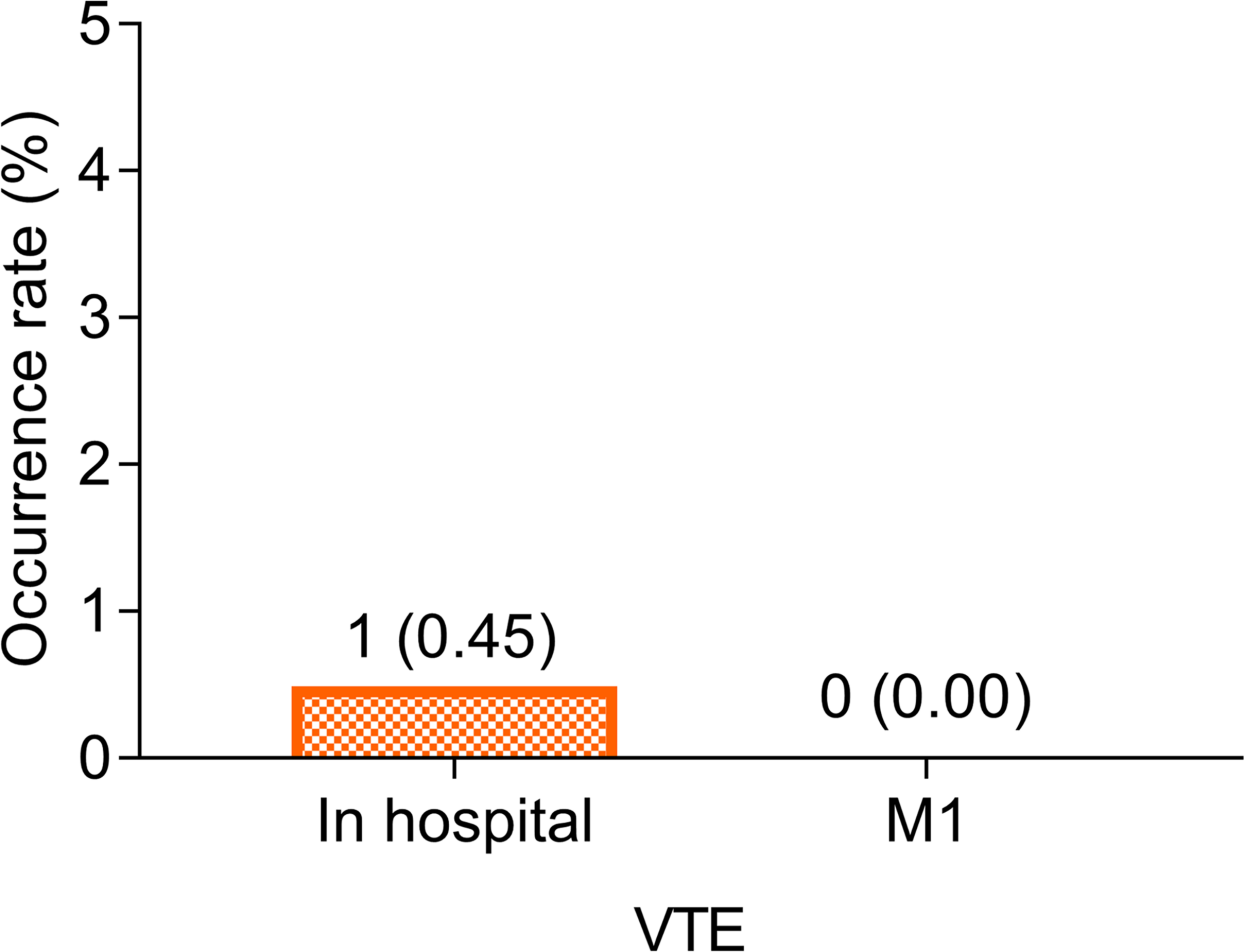
VTE rate in the hospital and at M1. VTE rate in the hospital and at M1 in cancer patients who received fondaparinux.

Only 1 (0.45%) patient had VTE in the hospital. However, the specific disease condition, treatment method, and treatment outcome of this patient were missing and could not be traced. Moreover, there was no (0.00%) VTE occurrence at M1 in cancer patients who received fondaparinux (**Figure 1**).

### Bleeding and death rate in fondaparinux-treated cancer patients

**Figure 2:**
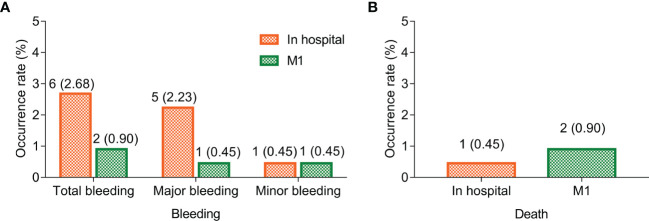
Bleeding and death rate in the hospital and at M1. Bleeding **(A)** and death **(B)** rate in the hospital and at M1 in cancer patients who received fondaparinux.

The bleeding rate was 2.68% in the hospital, among which the major bleeding rate was 2.23% and the minor bleeding rate was 0.45%. Moreover, the bleeding rate was 0.90% at M1, among which both the major and minor bleeding rates were 0.45% (**Figure 2A**). Notably, there was no bleeding event in the four aforementioned patients with a combination of aspirin. Furthermore, the death rates in the hospital and at M1 were 0.45% and 0.90% in cancer patients who received fondaparinux, respectively (**Figure 2B**). The cause of death of the patient in the hospital was hemorrhagic shock caused by rupture and bleeding of esophageal cancer. In addition, at M1, the cause of death of one of two patients was hemorrhagic shock caused by rupture and bleeding of esophageal cancer, while the cause of death of another patient was unknown.

### Bleeding occurrence in fondaparinux-treated patients with different cancer types

Major bleeding in the hospital occurred in one (0.45%) patient with gynecologic cancer, one (0.45%) patient with lung cancer, one (0.45%) patient with gastric cancer, one (0.45%) patient with esophageal cancer, and one (0.45%) patient with liver cancer. Major bleeding at M1 occurred in one (0.45%) patient with other cancers. Minor bleeding in the hospital occurred in one (0.45%) patient with esophageal cancer. Meanwhile, minor bleeding at M1 occurred in one (0.45%) patient with colorectal cancer (**Table 3**).

**Table 3:** Bleeding occurrence in patients with different cancer types.

Items	Major bleeding, n (%)		Minor bleeding, n (%)	
	In hospital	At M1	In hospital	At M1
Gynecologic cancer	1 (0.45)	0 (0.00)	0 (0.00)	0 (0.00)
Lung cancer	1 (0.45)	0 (0.00)	0 (0.00)	0 (0.00)
Colorectal cancer	0 (0.00)	0 (0.00)	0 (0.00)	1 (0.45)
Nasopharyngeal cancer	0 (0.00)	0 (0.00)	0 (0.00)	0 (0.00)
Gastric cancer	1 (0.45)	0 (0.00)	0 (0.00)	0 (0.00)
Esophageal cancer	1 (0.45)	0 (0.00)	1 (0.45)	0 (0.00)
Liver cancer	1 (0.45)	0 (0.00)	0 (0.00)	0 (0.00)
Pancreatic cancer	0 (0.00)	0 (0.00)	0 (0.00)	0 (0.00)
Lymphoma	0 (0.00)	0 (0.00)	0 (0.00)	0 (0.00)
Others	0 (0.00)	1 (0.45)	0 (0.00)	0 (0.00)

M1, 1 month after treatment.

### Types of bleeding in fondaparinux-treated cancer patients

Major bleeding in the hospital included one (0.45%) patient with fatal bleeding and four (1.78%) patients with hemoglobin declined ≥2 g/dl. Meanwhile, major bleeding at M1 included one (0.45%) patient with fatal bleeding. Moreover, minor bleeding in the hospital included one (0.45%) patient with blood-stained sputum, and minor bleeding at M1 included one (0.45%) patient with gingival bleeding (**Table 4**).

**Table 4:** Types of bleeding.

Types of bleeding	Patients (N = 224)
Major bleeding in hospital, n (%)	
Fatal bleeding	1 (0.45)
Hemoglobin declined ≥2 g/dl	4 (1.78)
Major bleeding at M1, n (%)	
Fatal bleeding	1 (0.45)
Minor bleeding in hospital, n (%)	
Blood-stained sputum	1 (0.45)
Minor bleeding at M1, n (%)	
Gingival bleeding	1 (0.45)

M1, 1 month after treatment.

### Association of platelet count with bleeding occurrence in fondaparinux-treated cancer patients

There was no relationship between platelet count and total bleeding in the hospital (*p* = 0.657), major bleeding in the hospital (*p* = 0.610), minor bleeding in the hospital (*p* = 1.000), total bleeding at M1 (*p* = 0.460), major bleeding at M1 (*p* = 0.265), or minor bleeding at M1 (*p* = 1.000) in fondaparinux-treated cancer patients (**Table 5**).

**Table 5:** Correlation of platelet count with bleeding occurrence.

Items	Platelet count		p-value
	Normal (n = 161)	Abnormal (n = 58)	
Total bleeding in hospital, n (%)			0.657
No	157 (97.52)	56 (96.55)	
Yes	4 (2.48)	2 (3.45)	
Major bleeding in hospital, n (%)			0.610
No	158 (98.14)	56 (96.55)	
Yes	3 (1.86)	2 (3.45)	
Minor bleeding in hospital, n (%)			1.000
No	160 (99.38)	58 (100.00)	
Yes	1 (0.62)	0 (0.00)	
Total bleeding at M1, n (%)			0.460
No	160 (99.38)	57 (98.28)	
Yes	1 (0.62)	1 (1.72)	
Major bleeding at M1, n (%)			0.265
No	161 (100.00)	57 (98.28)	
Yes	0 (0.00)	1 (1.72)	
Minor bleeding at M1, n (%)			1.000
No	160 (99.38)	58 (100.00)	
Yes	1 (0.62)	0 (0.00)	

M1, 1 month after treatment.

### Adverse events in fondaparinux-treated cancer patients

Of all cancer patients who received fondaparinux, 33 (14.73%) patients experienced adverse events. Specifically, there were seven (3.13%) patients with nausea and vomiting, five (2.23%) patients who had gastrointestinal reactions, three (1.34%) patients who experienced subcutaneous bleeding, three (1.34%) patients with reduced white blood cells, three (1.34%) patients who lost appetite, three (1.34%) patients who had fatigue, two (0.89%) patients who experienced fever, two (0.89%) patients who had a cough, and five (2.23%) patients who suffered from other adverse events (**Table 6**).

**Table 6:** Adverse events.

Items	Patients (*N* = 224)
Total, n (%)	33 (14.73)
Nausea and vomiting, n (%)	7 (3.13)
Gastrointestinal reactions, n (%)	5 (2.23)
Subcutaneous bleeding, n (%)	3 (1.34)
Reduced white blood cells, n (%)	3 (1.34)
Loss of appetite, n (%)	3 (1.34)
Fatigue, n (%)	3 (1.34)
Fever, n (%)	2 (0.89)
Cough, n (%)	2 (0.89)
Others, n (%)	5 (2.23)

## Discussion

VTE includes PE and DVT, which is one of the major health problems in the world (16, 17). As a type of anticoagulant, fondaparinux has been widely used to prevent VTE and is considered to have certain clinical advantages in cancer patients (18). For example, one research shows that the incidence of VTE is 2.5% in colorectal cancer patients who received fondaparinux, which proves that it is effective in VTE prevention (19). Another study finds that fondaparinux reduces VTE and major bleeding events compared with warfarin in cancer patients (14). However, the information on fondaparinux in preventing VTE in Chinese cancer patients is still lacking. Our study suggested that the in-hospital VTE rate was 0.45% while there was no VTE occurrence at M1 in cancer patients who received fondaparinux. This finding showed a good VTE prevention effect of fondaparinux. This might be due to the fact that fondaparinux had fast selective inhibition of factor Xa, thereby inhibiting thrombin formation and thrombus expansion (12, 20). Meanwhile, fondaparinux had predictable linear pharmacokinetics and a long half-life duration (21). Therefore, fondaparinux could prevent VTE well and last for a long time in cancer patients.

Furthermore, the bleeding events caused by VTE are also noteworthy. A previous study found that the incidence of major bleeding in patients with colorectal cancer after surgery who received fondaparinux was 1.7% (19). In our study, similar findings revealed that the rates of the bleeding event in cancer patients who received fondaparinux were 2.68% in the hospital and 0.90% at M1. Furthermore, the death rates were 0.45% and 0.90% in the hospital and at M1, respectively. These results illustrated that the risk of bleeding and death of cancer patients who received fondaparinux was acceptable, suggesting the safety of fondaparinux. Possible explanations were as follows: (1) Fondaparinux did not bind to platelet factor 4 and reduced the risk of thrombocytopenia; meanwhile, it also did not affect prothrombin time or activated partial thromboplastin time (22, 23). Thus, the incidence of bleeding events in cancer patients who received fondaparinux was acceptable. (2) As mentioned above, fondaparinux effectively balanced the occurrence of VTE and bleeding events in cancer patients, which were both positively related to mortality (24, 25). Therefore, cancer patients who received fondaparinux had a low risk of death.

At present, there were only a few studies reporting the incidence of adverse events in cancer patients who received fondaparinux. A previous study revealed that the incidence rate of adverse events was 10.9% during fondaparinux treatment in colorectal cancer patients undergoing laparoscopic surgery (19). This was similar to our results, which observed that the rate of adverse events in cancer patients who received fondaparinux was 14.73%. This might be because fondaparinux had a certain degree of anti-inflammatory effect (26, 27), which reduced other adverse events such as fever in cancer patients to some extent. This finding indicated that the safety of fondaparinux in the treatment of cancer patients with VTE was acceptable and no new adverse events were reported. However, more research was needed to confirm that.

Our study had several limitations: (1) Our study had a small sample size, and further study should include more cancer patients to verify the efficacy and safety of fondaparinux. (2) The follow-up period in our study was short, and a longer follow-up period was needed to further evaluate the risk of VTE, bleeding, death, and adverse events in cancer patients who received fondaparinux. (3) Our study did not include the control group. Future studies should enroll the control group to further verify the efficacy and safety of fondaparinux in preventing VTE in Chinese cancer patients.

In conclusion, fondaparinux yields a low rate of VTE and low risks of bleeding and death with tolerable adverse events in treating cancer patients.

## Data availability statement

The original contributions presented in the study are included in the article/supplementary material. Further inquiries can be directed to the corresponding author.

## Ethics statement

The studies involving human participants were reviewed and approved by Bozhou Hospital Affiliated to Anhui Medical University. The patients/participants provided their written informed consent to participate in this study.

## Author contributions

LW and GC contributed to the conception and data acquisition. ZS, CX, RL, WP, LX, and FC contributed to data analysis. LW, ZS, CX, RL, and GC drafted the manuscript. LW, WP, LX, FC, and GC revised the manuscript. All authors read and approved the final manuscript.
